# Dual role for the unfolded protein response in the ovary: adaption and apoptosis

**DOI:** 10.1007/s13238-016-0312-3

**Published:** 2016-09-08

**Authors:** Ning Huang, Yang Yu, Jie Qiao

**Affiliations:** Beijing Key Laboratory of Reproductive Endocrinology and Assisted Reproductive Technology and Key Laboratory of Assisted Reproduction, Ministry of Education, Center of Reproductive Medicine, Department of Obstetrics and Gynecology, Peking University Third Hospital, Beijing, 100191 China

**Keywords:** endoplasmic reticulum stress, obesity, ovary, follicle, ovarian diseases

## Abstract

The endoplasmic reticulum (ER) is the principal organelle responsible for several specific cellular functions including synthesis and folding of secretory or membrane proteins, lipid metabolism, and Ca^2+^ storage. Different physiological as well as pathological stress conditions can, however, perturb ER homeostasis, giving rise to an accumulation of unfolded or misfolded proteins in the ER lumen, a condition termed ER stress. To deal with an increased folding demand, cells activate the unfolded protein response (UPR), which is initially protective but can become detrimental if ER stress is severe and prolonged. Accumulating evidence demonstrates a link between the UPR and ovarian development and function, including follicular growth and maturation, follicular atresia, and corpus luteum biogenesis. Additionally, ER stress and the UPR may also play an important role in the ovary under pathological conditions. Understanding the molecular mechanisms related to the dual role of unfolded protein response in the ovarian physiology and pathology may reveal the pathogenesis of some reproductive endocrine diseases and provide a new guidance to improve the assisted reproductive technology. Here we review the current literature and discuss concepts and progress in understanding the UPR, and we also analyze the role of ER stress and the UPR in the ovary.

## Introduction

**Figure 1:**
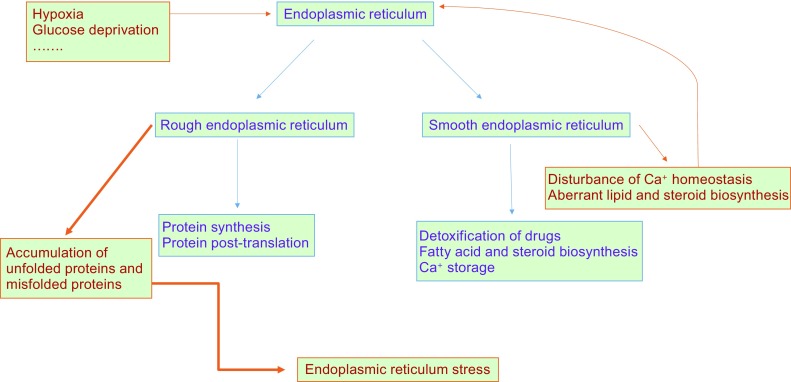
**Normal ER function and ER stress**. The ER has two main forms, namely the rough and smooth ER (RER and SER) according to their different structures and functions. RER is studded with abundant ribosomes and is mainly responsible for protein folding and post-translational modification. SER does not have attached ribosomes and is a primary site for drug detoxification, fatty acid and steroid biosynthesis, and Ca^+^ storage. Some physiological or pathological conditions, such as hypoxia and glucose deprivation, can perturb ER function, leading to accumulation of unfolded and misfolded proteins within the ER and resulting in induction of ER stress. In addition, perturbation of Ca^2+^ homeostasis and aberrant lipid and steroid biosynthesis may further impair ER function

The endoplasmic reticulum (ER) is a major compartment within eukaryotic cells responsible for synthesis and folding of proteins destined for the secretory pathway or insertion into the membrane, trafficking and metabolism of lipids and sterols, and cellular Ca^2+^ storage (Duan et al., 2015; Wang et al., 2015; Daniele and Schiaffino 2016). Some physiological or pathological conditions that perturb ER function—such as glucose deprivation (Marjon et al., 2004), hypoxia (Gao et al., 2016), aberrant Ca^2+^ regulation(Zhou et al., 2015), and elevated free fatty acid levels (Cui et al., 2013)—lead to accumulation of misfolded proteins within the ER, resulting in the induction of ER stress (Fig. 1).

**Figure 2:**
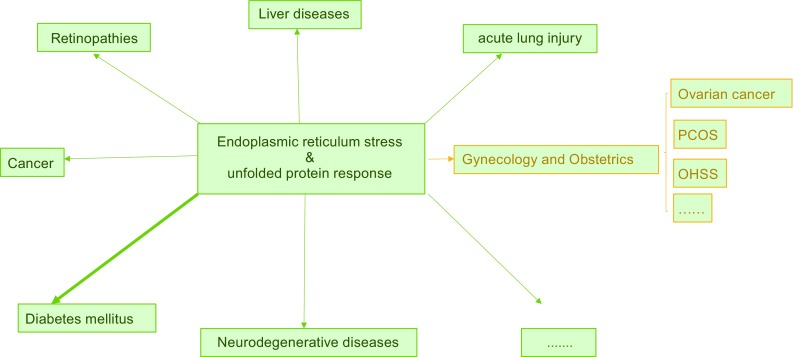
**UPR and related diseases**. Increasing evidence demonstrates the relationship between the unfolded protein response and diseases that involve different human organs. PCOS, polycystic ovarian syndrome; OHSS, ovarian hyperstimulation syndrome

To cope with ER stress, cells activate a series of protective intracellular signaling pathways, collectively termed the unfolded protein response (UPR). These include transient attenuation of translation to reduce the protein synthesis and folding load, activation of transcription of chaperone proteins and folding catalysts to expand the folding capacity of the ER, induction of ER-associated degradation (ERAD) to remove terminally misfolded proteins (i.e., that fail to fold into the correct native structure or assemble into proper protein complexes), and induction of apoptosis (Ron and Walter 2007; Olzmann et al., 2013). The UPR pathway elicits paradoxical outputs, inducing cytoprotective functions that reestablish homeostasis and cytodestructive functions that promote apoptosis, which is initially an effective means to eliminate a minority of cells that cannot recover from ER stress and protect the organism from damage induced by ER stress (Tabas and Ron, 2011; Iurlaro and Munoz-Pinedo 2016). Whether the UPR promotes cell survival or cell death depends on the duration and severity of the ER stress (Shore et al., 2011). The UPR can promote survival under conditions of transient and mild ER stress, or conversely promote cell death by activating downstream apoptosis signaling molecules if ER stress is prolonged and severe. Although cell death under severe ER stress may guard organisms from exposure to improperly folded proteins, many prevalent human diseases—such as diabetes mellitus (Back et al., 2012; Brozzi and Eizirik 2016), acute lung injury (Hu et al., 2015; Li et al., 2015), and retinopathies (Rana et al., 2014; Cai et al., 2015)—may be caused by excessive ER stress–induced cell death (Fig. 2).

The ovary has dual roles, i.e., reproductive function (responsible for the differentiation and release of mature oocytes for fertilization) and endocrine function (in charge of the synthesis and secretion of sex hormones, such as estrogen, progesterone and androgen). Follicles are the fundamental functional units of the mammalian ovary and consist of an innermost single oocyte, the surrounding granulosa cells (GCs) and the outer layers of theca interna cells, and theca externa cells. Beginning with the recruitment of primordial follicles from a reserve pool into the growing pathway, follicles develop through four major stages: primary, secondary, antral, and preovulatory (McGee and Hsueh 2000; Monniaux et al., 2014). Follicular growth is achieved through an increase in oocyte size accompanied by proliferation of the surrounding GCs. After the secondary follicular stage, the rate of oocyte growth and GC proliferation can increase progressively, which may result in a hypoxic condition (Monniaux et al., 1997; Monniaux et al., 2016). Such local conditions may contribute to ER dysfunction, leading to accumulation of unfolded and misfolded proteins that induce ER stress and the UPR (Harada et al., 2015). Under endocrine and paracrine control, one follicle is selected among a cohort of simultaneously growing follicles, and the other follicles enter follicular atresia (Scaramuzzi et al., 2011), which may be induced in part by ER stress (Lin et al., 2012). The corpus luteum develops from the follicle cells surrounding the ovulatory follicle and functions as a major site for the synthesis and secretion of progesterone. Some studies have indicated a link between the UPR and progesterone synthesis (Park et al., 2013a). In addition to their functions in ovarian physiology, ER stress and the UPR may also play a role in ovarian pathology.

## Canonical UPR: a bifunctional response causing cell survival or cell death

**Figure 3:**
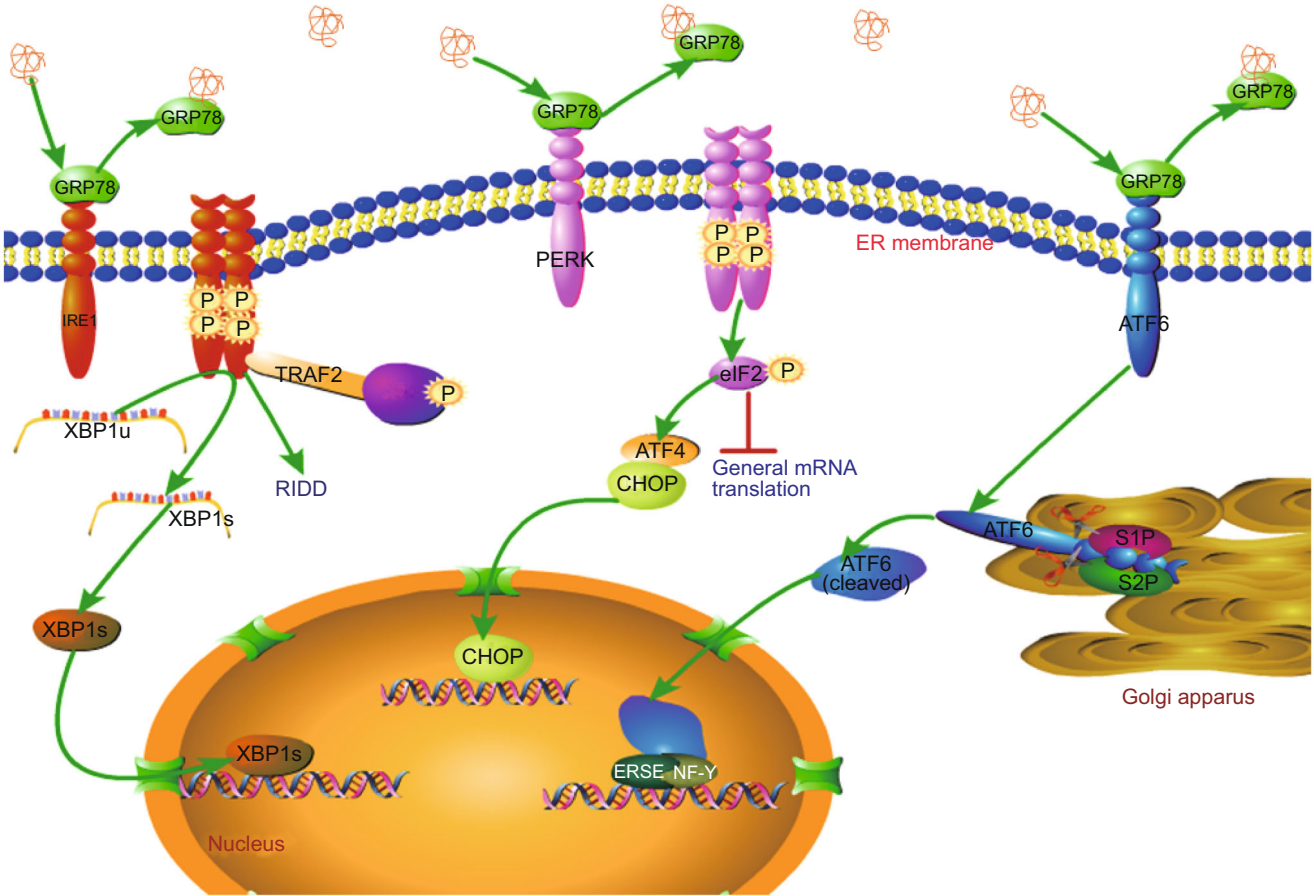
**Canonical UPR pathway**. The UPR is triggered by three ER stress sensors—PERK, IRE1α, and ATF6—which are rendered inactive by binding to GRP78 under non-stressed conditions. Progressive accumulation of misfolded proteins during ER stress results in dissociation of GRP78 from the GRP78-sensor complex, inducing dimerization of PERK and IRE1α as well as transport of ATF6 from the ER to the Golgi. IRE1α homodimerization and autophosphorylation initiates its kinase and endoribonuclease activities to generate an important transcription factor XBP1 s, which enters the nucleus to regulate the expression of a wide array of genes involved in ER function. Severe and chronic ER stress causes excess activation of the IRE1α kinase and RNase activities, leading to regulated IRE1α-dependent decay and phosphorylation of c-Jun terminal kinase (JNK). Similarly, PERK homodimerization and autophosphorylation induces phosphorylation of the α subunit of translation initiation factor 2 (eIF2α), which blocks formation of ribosomal preinitiation complexes and suppresses translation initiation, thereby attenuating general mRNA translation. PERK activation paradoxically promotes transcription initiation of the gene encoding ATF4 even when eIF2α is substantially phosphorylated, leading to upregulation of CHOP Once activated by ER stress, ATF6 moves to the Golgi, where it is cleaved by S1P and S2P proteases to generate an active form that mediates transcriptional induction of ER-localized molecular chaperones and folding enzymes

As a homeostatic signaling pathway, the UPR affords unfolded or misfolded proteins an additional opportunity to fold into their native state triggered by the interactions of three ER stress signaling sensors: protein kinase RNA (PKR)-like ER kinase (PERK), inositol-requiring enzyme 1α (IRE1α), and activating transcription factor 6α (ATF6α). In the absence of ER stress, these sensors bind to the ER-resident glucose-regulated protein 78 (GRP78) chaperones in their intraluminal domains (N-terminal portion of IRE1α and PERK and C-terminal portion of ATF6) and thereby render the sensors inactive (Bertolotti et al., 2000; Shen et al., 2005). Upon stress, accumulated unfolded or misfolded proteins are sensed by the luminal domain of transmembrane proteins, resulting in the dissociation of stable GRP78-sensor complexes (consisting of ATF6, IRE1α, PERK) and inducing oligomerization of PERK and IRE1α as well as transport of ATF6 from the ER to the Golgi (Bertolotti et al., 2000; Shen et al., 2005). The role of GRP78 as a central regulator of the UPR makes it a master marker for detecting the induction of ER stress (Lee, 2005). In addition, some researchers have found that PERK and IRE1α can directly sense unfolded proteins through their core ER luminal domain to activate downstream pathways (Gardner and Walter 2011) (Fig. 3).

### PERK pathway

PERK is a single-pass ER transmembrane kinase that is synthesized as a type I transmembrane glycoprotein (Haze et al., 1999). Upon activation by the accumulation of unfolded proteins in the ER lumen, PERK phosphorylates the Ser51 residue of the α subunit of translation initiation factor 2 (eIF2α) (Harding et al., 2000; Scheuner et al., 2001), which blocks formation of ribosomal preinitiation complexes and suppresses tion of translation, thereby decreasing the number of proteins translocated into the ER (Marciniak et al., 2006).

Although translation of most mRNAs is attenuated by limiting eIF2α, a subset of mRNAs is preferentially translated even with substantial eIF2α phosphorylation. In this way, PERK activation paradoxically promotes the transcription initiation of ATF4 (Harding et al., 2000) and induces ATF4 binding to the C/EBP-ATF composite site in the CHOP promoter to cause the transcription of the gene encoding CHOP (Ma et al., 2002). CHOP is a member of the C/EBP family of transcription factors and can induce apoptosis via several pathways (McCullough et al., 2001; Novoa et al., 2001; Marciniak et al., 2004; Yamaguchi and Wang, 2004; Lu et al., 2014).Some researchers have found that activation of TLR–TRIF signaling (e.g., by low dose of lipopolysaccharide) can selectively suppress ATF4-CHOP expression under prolonged ER stress, which may provide a promising therapeutic strategy for some degenerative diseases related to activation of CHOP (Woo et al., 2009).

### IRE1α pathway

IRE1α, the most ancient of the three signaling sensors, consists of an N-terminal luminal domain, a single-pass transmembrane-spanning segment, and a cytosolic region subdivided into a Ser/Thr protein kinase domain and a C-terminal endoribonuclease (RNase) domain (Calfon et al., 2002). It is synthesized as a type I transmembrane glycoprotein. (Haze et al., 1999). Accumulation of unfolded and misfolded proteins activates IRE1α and causes oligomerization of the N-terminal luminal domain (Credle et al., 2005). Serving as a bifunctional enzyme possessing both a protein kinase and a site-specific RNase, activation of IRE1α promotes autophosphorylation of its cytoplasmic kinase domain, which leads to homo-oligomerization of the kinase/RNase domains (Korennykh et al., 2008). This gives the RNase the ability to site-specifically splice XBP1 mRNA, removing a 26-base intron and generating a form of the mRNA that encodes a transcriptional activator protein to enhance ER folding capacity and quality control (Yoshida et al., 2001; Calfon et al., 2002; Ghosh et al., 2014).

Under acute ER stress, IRE1α induces an adaptive response that promotes cell survival. However, chronic ER stress causes increased phosphorylation of the IRE1α kinase, proportionally increasing the oligomeric state of the kinase/RNase subunits past a critical threshold, thereby driving RNase activity into a hyperactive state and relaxing its substrate specificity to endonucleolytically cleave many other mRNAs. Consequently, high RNase activity leads to endonucleolytic decay of ER-localized mRNAs (Han et al., 2009) and activation of thioredoxin-interacting protein (Ghosh et al., 2014). Furthermore, increased oligomerization and activation of kinase/RNase subunits are sufficient for initiating the phosphorylation/activation of c-Jun terminal kinase (JNK) via clustering of the N-terminal effector domain of TRAF2, an adaptor protein that is bound to the C-terminal cytoplasmic protein IRE1 and couples the plasma membrane receptor to JNK activation (Urano et al., 2000).

The UPR can activate the mitochondrial apoptotic pathway, which is critically regulated] by several members of the Bcl-2 family. This family can be functionally classified as anti-apoptotic (i.e., Bcl-2, Bcl-XL, Bcl-w) and pro-apoptotic (i.e., Bad, Bid, Bik, Bim, Bad) (Martinou and Youle, 2011). Some studies have demonstrated that ER stress induces conformational changes in Bax and Bak, changing them from inactive to active forms and setting the apoptotic pathway in motion (Zong et al., 2003). The ATF4-CHOP pathway can downregulate the expression of Bcl-2 (McCullough et al., 2001), and the IRE1α-JNK pathway can lead to phosphorylation and subsequent inactivation of Bcl-2 and Bcl-XL (Fan et al., 2000). Taken together, these results suggest that the Bcl-2 family plays a crucial role in regulating ER stress–induced apoptosis.

### ATF6α pathway

ATF6 is synthesized as a type II transmembrane glycoprotein and is embedded in the ER membrane (Haze et al., 1999). It has a luminal domain, a single transmembrane domain, and a cytoplasmic domain consisting of an N-terminal bZIP domain and a transcriptional activation domain (TAD). When unfolded proteins accumulate in the ER, the CD1 region of the luminal domain senses this ER stress and provides a positive signal for ATF6 translocation from the ER to the Golgi (Chen et al., 2002; Sato et al., 2011), in which reside the site 1 and site 2 proteases (S1P and S2P, the enzymes that process SREBPs in response to cholesterol deprivation) (Ye et al., 2000). ATF6 is cleaved in a two-step process in the Golgi, first by S1P in the luminal domain (near amino acid 418) and then by S2P near the junction between the cytoplasmic and transmembrane domains, allowing dissociation of the N-terminal bZIP and TAD (Ye et al., 2000). The TAD then moves to the nucleus, where it activates transcription of target genes by recognizing and directly binding the ER stress element (ERSE) in cooperation with the transcription factor NF-Y, which is also bound to the ERSE (Yoshida et al., 1998; Yoshida et al., 2000). ATF6-mediated transcriptional induction of ER-localized molecular chaperones and folding enzymes can improve the ER folding capacity, contributing to the maintenance of ER homeostasis in mammals (Yoshida et al., 1998). In addition to promoting expression of chaperones, cleaved ATF6 also transactivates CHOP and XBP1 by binding to the ERSE together with NF-Y, which initialized a pathway that converges with the PERK/IRE1α pathway (Yoshida et al., 2000; Ma et al., 2002; Guo et al., 2014).

## Activation of the UPR in the ovarian cycle

As mentioned above, the follicle is the fundamental functional unit of the ovary. The ovarian cycle describes the normal changes that occur in the follicles and can be divided into three phases: follicular, ovulation, and luteal.

### Follicular growth and maturation

Recent evidence suggests the involvement of the UPR in the physiology of follicular growth and maturation. XBP1 s and HSPA5 (also known as GRP78) are expressed in GCs of late-stage follicles (i.e., at larger secondary, antral and pre-ovulatory stages), and this expression is accompanied by activation of IRE1 and PERK (Harada et al., 2015). During follicular growth after the secondary follicle stage, more protein must be synthesized to support proliferation of GCs. Over-loaded proteins] in the ER can induce ER stress and subsequently induce UPR pathways Some findings have demonstrated that the levels of XBP1 s mRNA in CC(cumulus cells) enclosingoocytes that achieve fertilization were higher than those in CC enclosing oocytes without the capacity for fertilization, and this may be related to the pro-angiogenic role of XBP1 (Calfon et al., 2002; Zeng et al., 2013; Harada et al., 2015).

Follicle-stimulating hormone (FSH) is the major stimulator of follicle growth and development in the final and preantral stages (Oktay et al., 1997; Hsueh et al., 2015; Babayev et al., 2016). FSH attenuates ER stress in mouse GCs in vivo and in vitro (Babayev et al., 2016). Conversely, induction of severe ER stress inhibits the FSH response and negates the effects of FSH on GCs expressed ER stress-associated gene (Babayev et al., 2016).

### Follicular atresia

In each reproductive cycle, only a limited number of follicles in the mammalian ovary undergo maturation and ovulation, whereas most follicles undergo a degenerative process known as atresia. Many studies have demonstrated that follicular atresia is predominantly regulated by GC apoptosis (Asselin et al., 2000). Severe and persistent ER stress will overwhelm adaption mechanisms, causing the UPR to initiate apoptosis. Thus, it is possible that ER stress plays an important role in regulating GC apoptosis. The protein GRP78 is present in GCs of non-atretic and atretic follicles of goat ovaries, but levels are higher in atretic follicles than in non-atretic ones. In contrast, CHOP is detected in GCs of atretic follicles but not in those of non-atretic follicles. Furthermore, the localization of GRP78 and DDIT3 on the antral side of the granulosa layer of atresia follicles (Lin et al., 2012) is similar to the localization of apoptotic cells in atretic follicles of goat ovaries (Bhardwaj and Sharma, 2011). ATF6 and ATF4 mRNAs are also increased during GC apoptosis (Lin et al., 2012). In addition, spontaneous apoptosis is also observed in vitro in GCs exposed to tunicamycin and serum withdrawal treatment through the activation of ER stress (Lin et al., 2012), which further demonstrates the crucial role of the UPR in the regulation of GC apoptosis. The low level of GRP78 found in healthy follicles indicates that GRP78 may play an important role in GC proliferation (Luo et al., 2006) and follicular development, and the high level of GRP78 and DDIT3 found in atretic follicles demonstrates that ER stress-induced apoptosis may act in the regulation of selective GC apoptosis in goat ovaries (McCullough et al., 2001; Novoa et al., 2001; Marciniak et al., 2004; Yamaguchi and Wang 2004; Woo et al., 2009).

Death ligand-receptor and mitochondria-mediated apoptotic systems have been shown to be active in GCs of mammalian ovaries (Manabe et al., 2008; Zhang et al., 2015a). However, several signaling molecules involved in these systems (including JNK, Bcl-2, Bax and TRAF) are also present in the UPR and induce apoptosis (Urano et al., 2000). Cross-talk between these two pathways and the UPR indicates that the ER stress system may play an indirect role through interaction with death ligand-receptor and mitochondria-mediated molecular signaling pathways in the regulation of GC apoptosis.

### Corpus luteum biogenesis

The corpus luteum is a transient endocrine organ derived from ovulated follicles (Devoto et al., 2009). The main function of the corpus luteum is to synthesize and secrete progesterone, which is indispensable for maintenance of pregnancy and regulation of the estrous cycle (Stouffer 2003; Devoto et al., 2009). The luteal phase can be divided into three main stages, namely development, maintenance, and regression, according to the macroscopic appearance of corpus luteum tissues (Stouffer et al., 2013). The pathway for progesterone synthesis mainly involves three steroidogenic enzymes, namely steroidogenic acute regulatory protein (known as StAR), p450 cholesterol side-chain cleavage enzyme (p450), and 3β-hydroxysteroid dehydrogenase, and two cellular organelles (mitochondria and ER) (Niswender 2002; Rekawiecki et al., 2008). As mentioned previously, the ER is important not only for protein synthesis and lipid metabolism but also for steroid hormone synthesis. Therefore, ER stress and the UPR may play a significant role in regulating the production of progesterone and the development of the corpus luteum. All three UPR signaling pathways, including adaptive or apoptotic signaling molecules (Grp78, p-eIF2α, ATF4, CHOP, ATF6, p-IRE1α, XBP1 s and p-JNK), are activated during luteal-phase progression during the estrous cycle of bovines] (Park et al., 2013a) and mice (Park et al., 2014). Pro-apoptotic signaling molecules such as cleaved caspase3, JNK, and CHOP were detected, and JNK activation and CHOP expression via ER stress–mediated pro-apoptotic signaling cascades occurred prior to caspase3 activation during the regression stage of the corpus luteum (Urano et al., 2000; McCullough et al., 2001; Novoa et al., 2001; Marciniak et al., 2004; Yamaguchi and Wang 2004; Woo et al., 2009; Park et al., 2013a; Park et al., 2014). Furthermore, the dynamic change of these molecules seems to have some connection with the steroidogenic enzymes, which suggests that the UPR may influence the expression of steroidogenic enzymes (Park et al., 2013a; Park et al., 2013b). Molecules involved in UPR signaling, as activated during different stages of the luteal phase, may be related to progression of the luteal phase.

In conclusion, ER stress and the short-term UPR as an adaptive response are beneficial and necessary in the physiology of follicular growth and maturation.

## Obesity and aberrant activation of the UPR in the ovary

Obesity has become a worldwide public health concern, as it is a major factor leading to development of insulin resistance, type 2 diabetes, fatty liver disease, and some cancers (Grundy 2015; Font-Burgada et al., 2016; Baidal and Lavine 2016). Obesity impairs oocyte function and causes alterations in the follicle and oocyte, which induces various types of reproduction dysfunction, including reduced conception, infertility, and early pregnancy loss (Hou et al., 2016; Sessions-Bresnahan et al., 2016). It is well established that obesity is closely connected with ER stress and the UPR.

The lipid composition of the ER membrane is unique, as the cholesterol content is particularly low (Feng et al., 2003) and unsaturated phosphatidylcholine is the major phospholipid (Leamy et al., 2013). This special membrane composition allows the ER to maintain a high degree of fluidity that facilitates its function. Abnormal incorporation of free cholesterol or saturated phospholipid species can result in detrimental stiffening of cellular membranes and loss of function. A large amount of free cholesterol trafficking to the ER of macrophages increases cholesterol-induced lipid ordering of the ER membrane and perturbs membrane protein conformation and function, providing a plausible mechanism for the decrease in SERCA activity induced by loading of the membrane with free cholesterol (Li et al., 2004). In addition, a recent study showed that the increased phosphatidylcholine: phosphatidylethanolamine ratio found in the ER of obese liver (both genetically induced and diet-induced) significantly inhibits SERCA function (Fu et al., 2011). The ER lumen is the major intracellular site for Ca^2+^ storage and release and provides a unique environment with a high concentration of Ca^2+^-binding proteins. This directly influences the function of the ER, affecting its roles in protein and sterol synthesis, lipid metabolism, and signal transduction (Coe and Michalak, 2009; Krebs et al., 2015). Inhibition of SERCA causes depletion of ER Ca^2+^ stores and disruption of ER Ca^2+^ homeostasis, leading to induction of ER stress and the UPR to control damage and re-establish ER homeostasis (Guerrero-Hernandez et al., 2014).

Increased body mass index in women is associated with elevated triglycerides, insulin, and free fatty acids in ovarian follicular fluid. (Pantasri et al., 2015; Robker et al., 2009). Treatment of mouse oocytes with this lipid-rich follicular fluid impairs oocyte maturation and induces ER stress and the UPR (Robker et al., 2009; Yang et al., 2012; Pantasri et al., 2015). Another study directly demonstrated that a high-fat diet dramatically increases the lipid content in mouse oocytes, both before and after ovulation, and induces ER stress pathway genes, alters mitochondrial membrane potential, and increases the incidence of apoptosis in ovarian cells. The UPR marker ATF4 is specifically upregulated in GCs and COCs (cumulus-oocyte complex)isolated from pre-ovulatory follicles of mice given a high-fat diet (GRP78 is also upregulated in COCs). ATF4 is also upregulated in the GCs of obese women (Wu et al., 2010). Compared with mice on a control diet, mice given a high-fat diet have higher rates of anovulation and lower rates of fertilization (Wu et al., 2010).

Palmitic acid, a saturated fatty acid that is a physiological component in human follicular fluid, is a potential physiological inducer of ER stress (Danino et al., 2015; Haywood and Yammani, 2015). Exposure of COCs to a high dose of palmitic acid can induce ER stress and the UPR, accompanied with reduced secretion of PTX3 (a protein marker for oocyte development and fertilization; (Zhang et al., 2005; Baranova et al., 2014), mitochondrial dysfunction and impaired oocyte maturation, and fertilization. These results are similar to those observed in COCs treated with the classical ER stress inducer thapsigargin (Wu et al., 2012). Notably, each of these defects is restored with the ER stress inhibitor salubrinal, further demonstrating that ER stress is a key mechanism mediating fatty acid–induced defects in oocyte and developmental potential (Wu et al., 2012).

## The UPR and ovarian diseases

As an intracellular defense mechanism to attenuate ER stress and maintain organism homeostasis, the UPR is involved in the pathogenesis] of numerous human diseases, including ovarian diseases. Recent studies have shown a positive correlation between spliced XBP1 (induced by ER stress) and ovarian hyperstimulation syndrome (OHSS), a major complication during infertility treatment that is mainly characterized by increased capillary permeability (Elchalal and Schenker, 1997; Takahashi et al., 2016). Compared with patients without OHSS, follicles from OHSS patients express higher levels of XBP1 s in cumulus cells, which partly mediates upregulation of VEGFA, a key molecule in angiogenesis in the ovary that is regarded as a major cause of OHSS induction (Kosaka et al., 2007; Takahashi et al., 2016).

In primary solid tumors, the UPR is activated as a result of aberrant regulation of protein synthesis in cancer cells and changes in the tumor microenvironment, such as hypoxia, nutrition deprivation, and low pH (Brown and Giaccia, 1998; Giampietri et al., 2015). Cancer cells have the unique ability to exploit the UPR and its capacity to promote survival and growth. The UPR-related proteins GRP78and ATF6 are highly expressed in cancer cells and are involved in promoting cancer cell proliferation and survival under extreme conditions (Fu et al., 2007; Ye et al., 2010). Spliced XBP1 is a crucial factor for stimulating cancer progression by promoting tumor cell survival and metastatic potential and driving dendritic cell dysfunction to further inhibit anti-cancer immunity (Chen et al., 2014; Cubillos-Ruiz et al., 2015). An increasing number of studies have investigated similar roles the UPR may play in ovarian cancer and explored the potential for developing a promising target for therapy (McLean et al., 2009; De Carolis et al., 2014; Cubillos-Ruiz et al., 2015; Zhang et al., 2015b).

## Conclusions

The UPR, induced by ER stress, is an indispensable response for cells to restore cellular homeostasis; conversely, it may contribute to cell death when over-activated. The molecular pathways involved in the UPR have been clearly elucidated. Accumulating evidence suggests that ER stress and the UPR play a significant role in regulating ovarian structure and function, but our understanding of the molecular mechanisms involved remains incomplete. Future studies should further focus on the molecular mechanisms related to the dual role of the UPR in ovarian physiology and pathology to better understand changes in the ovarian cycle and provide new guidance to improve assisted reproductive technology.
